# Social Determinants of Oral Health: State-Level Analysis of Edentulism in Older Adults

**DOI:** 10.3390/healthcare14183022

**Published:** 2026-09-15

**Authors:** Simon Medcalfe, Catherine P. Slade, Lexie Bailey

**Affiliations:** 1 James M. Hull College of Business, Augusta University, Augusta, GA 30912, USA; 2 College of Education and Human Development, Augusta University, Augusta, GA 30912, USA

**Keywords:** edentulism, social determinants of oral health, dental health outcomes for older adults, health policy for dental health outcomes, dental professional role in smoking cessation

## Abstract

Background: The US has state variation in edentulism, commonly known as total tooth loss, in older adults. This paper identifies key social determinants of health (SDOH) associated with state differences in older adult edentulism that can be addressed with partnerships between dental providers and policymakers. Methods: Data was collected from three publicly available data sets, including County Health Rankings, provided by the University of Wisconsin Population Health Institute, the U.S. Centers for Disease Control and Prevention (CDC), and the Centers for Medicare and Medicaid Services (CMS). Results come from multiple regression analysis of panel data for all states and the District of Columbia for even years of 2012–2020. Results: A variety of SDOH, population health behaviors, and demographics are associated with older adult edentulism, with the largest effects for education and smoking. We have other variables of interest, and overall, our model explains over 80 percent of the variation. Conclusion: Policymaking in states should emphasize both education and smoking cessation programs for individuals. Future research may also consider the relationship between state-regulated dental insurance and dental practice location. States may also consider policies to encourage better dental professional training and incentives to address smoking cessation for patients.

## 1. Introduction

The relationship between oral health and overall health has been explored in detail for many years [1,2]. Poor oral health has been shown to be related to several types of less than desirable living conditions including unemployment, limited educational attainment, and high costs of medical and oral healthcare [3,4,5,6,7]. Similarly, social determinants of health (SDOH), or the conditions under which people are born and live [8] are associated with poor oral health, including dental utilization [9,10], extensive gum disease, and tooth loss [11].

A current national priority is refining policymaking to improve care coordination and reduce the burden of poor oral health [12,13]. The many policy decisions concerning allocation of resources to address the complex interplay of living conditions and health outcomes require geospatial analysis and ecological study [13], as a wide variety of federal and state SDOH initiatives continue to evolve [7]. These types of geospatial studies can help identify priorities for geographic areas, such as states or localities, that are facing a changing geopolitical landscape, including funding allocation to public health programs as well as insurance regulation [7,8].

**Figure 1:**
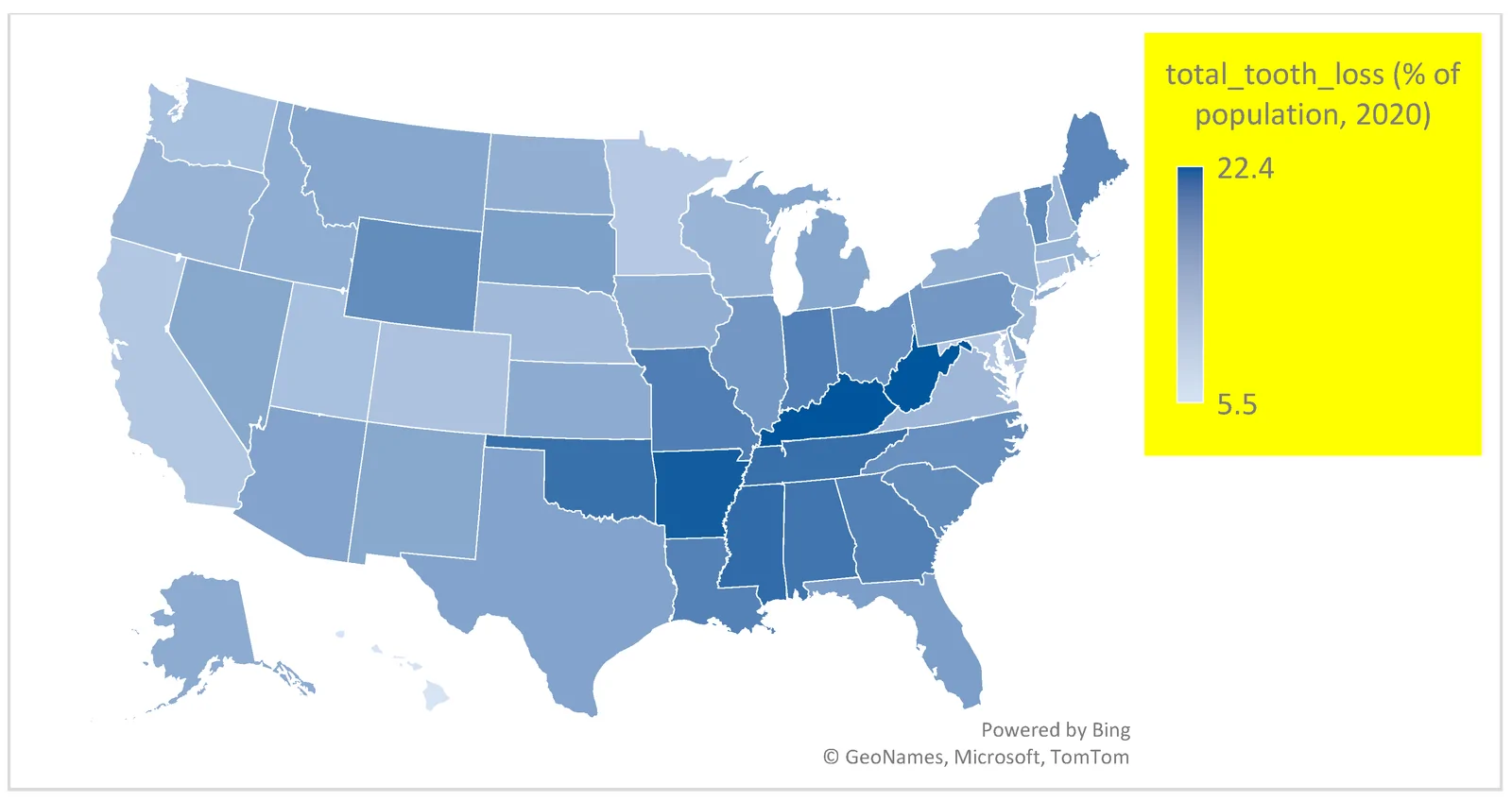
Percentage of the population with total tooth loss by state in 2020 (source: [16]).

One focus of poor oral health is edentulism, or total tooth loss, as it is associated with an individual’s mental health, such as poor self-image, as well as several associated diseases and chronic clinical conditions [14,15]. According to the U.S. Centers for Disease Control and Prevention (CDC) in 2020, an estimated 13.3% of adults aged 65 plus in the United States (U.S.) have lost all their natural teeth [16]. Although this number has declined from 16.2% in 2012, variations by state remain. For example, the average rate of edentulism in adults aged 65 or over between 2012 and 2020 was only 6% in Hawaii, while it was 29% in West Virginia. Figure 1 shows the visual geographic distribution of total tooth loss across the states.

Twenty-five years ago, the Surgeon General stated that the reason for these differences by state was unknown [1]. The follow-up report [6] continued to highlight edentulism differences by state. The main research objective of this paper is to identify the state-level characteristics that account for key differences in edentulism to educate and inform policymakers and practitioners.

Many studies have examined disparities in edentulism at the individual level [17,18,19,20]. These studies have identified individual-level characteristics such as socioeconomic status, health behaviors such as smoking and diabetes, access to dental care, and demographics such as race and ethnicity to be associated with edentulism [17,18,19,20]. However, these individual-level measures may be insufficient to fully explain the differences in total tooth loss at the state level, as there are many state-level policies that affect individual use of dental services [15]. We use an ecological study approach with state-level measures of total tooth loss and characteristics that are hypothesized to be associated with edentulism. The state is an important unit of measure because many health policies, such as Medicaid and Medicare coverage, are determined at the state level.

The SDOH framework [8] has been used to explore a variety of health outcomes. The SDOH framework categories, including economic stability, education, physical environment, social and community context, and the healthcare system, can be used to highlight specific priorities for study. The categories have some correspondence with the individual measures highlighted earlier, but they are measured at a geopolitical level. Studies of edentulism using the SDOH framework at the geopolitical level are fewer in number than individual-based studies and geopolitical level studies have mostly been conducted at the sub-state level, such as county [19] and census tract [15]. For example, Bastos et al. (2023) [20] used state-level structural racism and sexism variables to complement individual characteristics in edentulism. However, none of these studies attempted to explain the variation in edentulism between the states, the objective of our research. Additionally, our analysis includes variables that have been found to be important at the individual level but have not been examined at a geographic level, specifically smoking and diabetes prevalence [17,18,19]. We utilize a longitudinal panel model in contrast to prior studies that have primarily been cross-sectional for a limited number of years [15,20].

Our research includes variables representing a variety of SDOH categories, health behaviors, and state demographics. We also include dental service utilization and dental insurance coverage because these variables are both complicated from a policy standpoint, changing in recent years, and understudied [21,22,23]. For example, the 2023 Dental Benefits Report: Enrollment by the National Association of Dental Plans [21] shows an increase in recent years in publicly funded dental benefits, increasing 22.4% in 2022 compared to 2021.

Our testable hypothesis is that SDOH variables are correlated with state-level variation in edentulism in older adults.

## 2. Materials and Methods

We adopted the SDOH framework to answer the following research question:

What is the relationship between state-level edentulism in older adults and social determinants of health?

This is an ecological study using state-level data, including Washington DC, for even years 2012 through 2020. Data was collected from three publicly available data sets, University of Wisconsin County Health Rankings (accessed 6 September 2023) [24], Oral Health Data from the U.S. Centers for Disease Control and Prevention (https://www.cdc.gov/oral-health-data-systems/oral_health_data/oral-health-dashboard.html; accessed 20 September 2023) [16], and Medicaid Advantage Geographic Variation—National and State (2025) from the Centers for Medicare and Medicaid Services (https://data.cms.gov/summary-statistics-on-use-and-payments/medicare-geographic-comparisons/medicare-advantage-geographic-variation-national-state/data; accessed 30 May 2025) [25].

Our dependent variable is the state percentage of adults aged 65 and older who have lost all their natural teeth [16], one of the few dental conditions for which state-level data exists [1]. Our choice of state-level independent variables is informed by the literature, including the National Institutes of Health Oral Health in America: Advances and Challenges Report 2021 [6]. The report identifies “social determinants of health as crucial underlying factors that contribute to oral health disparities and inequities in the United States” (p. 7).

We include two independent variables for access to dental care. From the Oral Health Data Report [16], we determine the percentage of adults aged 18+ who have visited a dentist or dental clinic in the past year, pct_visited_dentist. From the University of Wisconsin Population Health Institute [24], we obtain the dentist_rate, or number of dentists per 100,000 population in each state.

Lacking a good source of dental insurance coverage by state population and year, we utilize two different variables to proxy for a patient’s ability to pay. Blackwell et al. (2019) [26] provide private dental coverage data by census regions of the U.S. for 2014–2017. This data identifies the percentage of dentate adults aged 18–64 who had dental care coverage (pct_dental_insurance). Coverage varies from 57.1% in New England to 45.6% in the South Atlantic and East South-Central regions. Unfortunately, the source data aggregates the 2014–2017 data into one value which we assign to all years. The second source is the percentage of the state population that is covered by Medicare Part C (pct_medicareC), which for some private plans include dental coverage [25]. Unfortunately, our data does not let us know whether a citizen has bought such a plan. Additionally, the data is only available for years 2016–2021.

All other variables come from the University of Wisconsin Population Health Institute [24]. We obtain measures of economic stability (median household income, income) and education (percentage of the population with at least some college education, pct_some_college) as indicators of socioeconomic status. For population health behaviors, we use the percentage of the population who are smokers (pct_smoking) and the percentage who have diabetes (pct_diabetes). Given growing interest in oral health disparities [6], we summarize the state population demographics including pct_black, pct_white, pct_hispanic, pct_female, and the percentage of the state that is rural (pct_rural). We include a linear time trend to account for the decline in edentulism over time. Table 1 indicates the source, population, geography, and available years for our data.

Our panel data is used to estimate multiple linear regression models using random effects. We have 255 observations (51 states over 5 years), except when using pct_medicareC, which is only available for three years. We use random effects over fixed effects because some of our insurance coverage data does not vary across the time period. We report robust confidence intervals based on robust standard errors clustered at the state level that adjust for the presence of heteroskedasticity. Multicollinearity among the explanatory variables was assessed and the correlations are shown in Table 2 along with the means and standard deviations. Some of our independent variables are obviously correlated. We checked for multicollinearity by calculating Variance Inflation Factors (VIFs). We never obtained high (VIF > 10) multicollinearity, with our highest VIF being 5.78 for percent black. The regression analyses were conducted using Stata 18 and 19.

## 3. Results

Tooth loss averages 16.5% across all states for even years 2012 through 2020. The maximum is 33.6% for West Virginia in 2014 and the minimum is 5.3% for Hawaii in 2012. Total tooth loss across all states generally shows a decline over time from 16.2% in 2012 to 13.3% in 2020.

The regression results for the three models are presented in Table 3. The first model, column 1, includes pct_medicareC; the second model, column 2, includes pct_dental_insurance; and the third model, column 3, includes both pct_medicareC and pct_dental_insurance. Across all three models, the percentage of the state population that has visited a dentist in the last year is negatively associated with complete tooth loss. The models suggest a wide variation in the magnitude of the effect. The midpoint estimates suggest that a one-percentage-point increase in the percentage of the population that has visited a dentist in the last year is associated with a 0.18 (model 1) to 0.02 (model 2) percentage-point lower edentulism rate. The dentist rate is positive; a ten-point increase in the dentist rate is associated with a 0.6 percentage-point increase in complete tooth loss. We fail to reject the null hypothesis that, on its own, medicareC is not associated with complete tooth loss. However, dental insurance is negatively associated with complete tooth loss; a one-percentage-point increase in dental insurance is associated with a 0.18 percentage-point lower population with complete tooth loss. Model 3, which includes both medicareC and dental insurance, indicates that the greater the percentage of the state population that has some form of dental insurance coverage, the lower the state edentulism.

Analyzing the SDOH variables, we find that median household income is unrelated to edentulism. The percentage of the state population that has some college education is negatively associated with complete tooth loss across all three models. The magnitude of the effect is large; a one-percentage-point increase in the state population that has some college education is associated with a lower edentulism rate of between 0.23 and 0.33 percentage points. The percentage of the state population that are smokers is positively associated with complete tooth loss across all three models. A one-percentage-point increase in smoking rates is associated with a 0.2 to 0.3 percentage-point increase in complete tooth loss. Of the demographic variables, only percent white is positively associated with complete tooth loss across all three models. The percentage of the population with diabetes is positively associated with edentulism at the 90% confidence level in model 2 (which includes dental insurance). The overall R-squared suggests that our models explain about 80 percent of the variation in state edentulism. The between R-squared is higher (0.88–0.91 depending on the model) than the within R-squared (0.19–0.21), indicating our model is better at explaining differences between states than within states.

In a robustness check, we run a correlated random effects (CRE) model to account for the possibility that unobserved state characteristics are correlated with the explanatory variables. The results are presented in Table 4. None of the fixed effects (FE) coefficients are significant, indicating that short-term changes within states are less important than long-term structural differences between states. Alternatively, it may be that the limited number of years in our analysis may reflect limited within-state level variation. Significant between-state effects are observed for pct_visited_dentist (β = −0.249, *p* = 0.021), dentist_rate (β = 0.070, *p* = 0.013), pct_some_college (β = −0.187, *p* = 0.009), pct_smoking (β = 0.316, *p* = 0.066), pct_white (β = 0.095, *p* = 0.000), and pct_female (β = 0.921, *p* = 0.071). The Mundlak test suggests that the random effects assumptions may not hold.

## 4. Discussion

**Figure 2:**
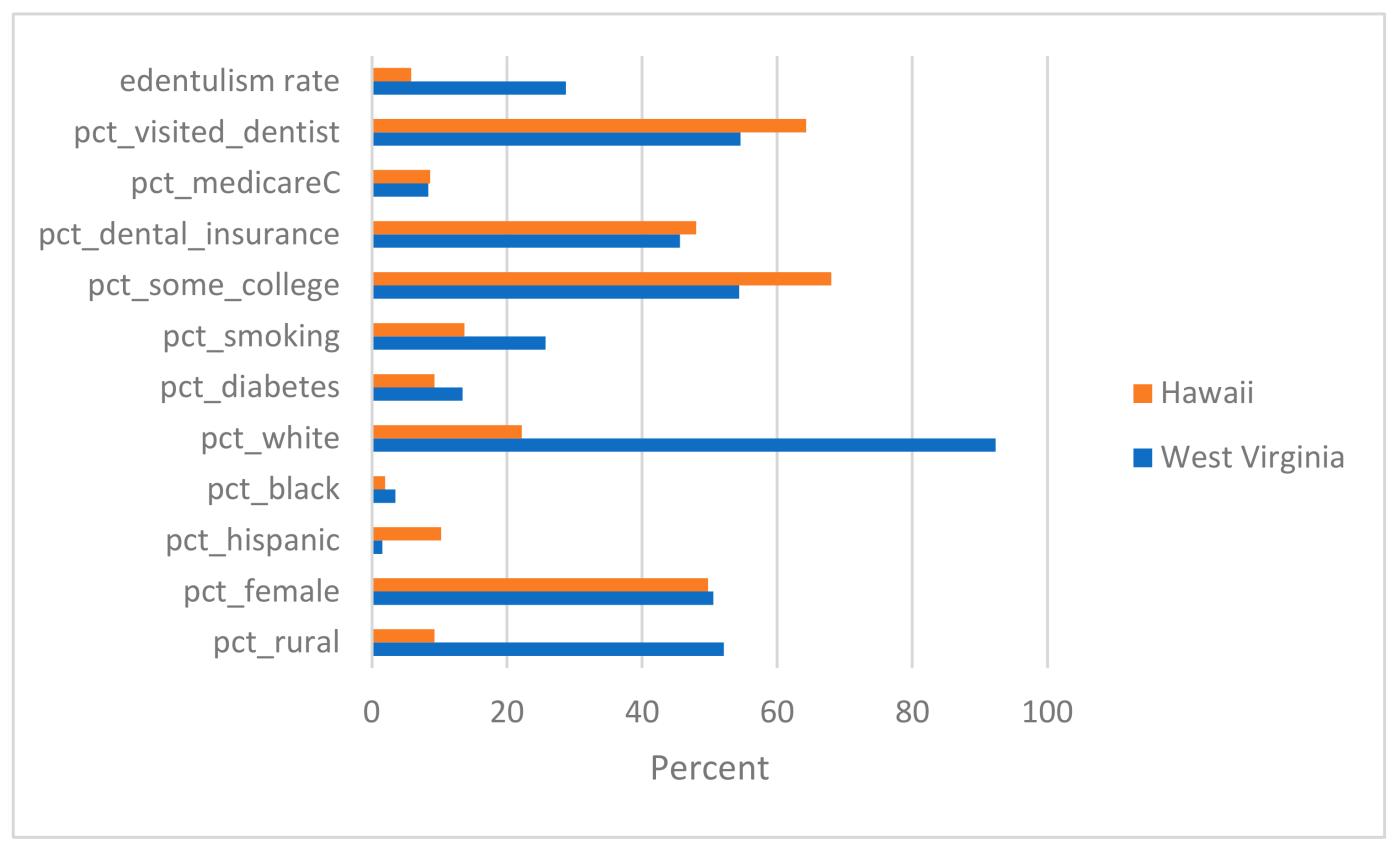
Comparison of Hawaii and West Virginia edentulism rate averaged over even years 2012 through 2020. Sources: Medicaid Advantage Geographic Variation—National and State [25]; University of Wisconsin Population Health Institute [24]; Blackwell et al. [26].

Our models are useful in answering our research question: why do edentulism rates for older adults vary across states? The average edentulism rate was lowest in Hawaii for 2012 through 2020 at 6 percent and highest in West Virginia at 29 percent during the same period. Figure 2 compares the data for Hawaii and West Virginia for our explanatory variables averaged over the five available years. The percentage of the state population that has visited a dentist in the last year is 64 percent in Hawaii and 55 percent in West Virginia. Hawaii has slightly higher dental insurance coverage, 48 percent compared to 46 percent in West Virginia. In total, 68 percent of the Hawaiian population has some college education but the rate is only 54 percent in West Virginia. Only 14 percent of the Hawaiian population smokes compared to 26 percent in West Virginia. The prevalence of diabetes is higher in West Virginia (13%) than Hawaii (9%). West Virginia has a higher percentage of Black and White residents (3% and 92%, respectively) compared to Hawaii (2% and 22%). In total, 51% of the state population of West Virginia is female compared to 50% in Hawaii. Only 9 percent of Hawaii’s population is considered rural compared to 52 percent of West Virginia. In addition to the data shown in Figure 2, Hawaii has a higher income than West Virginia ($75,540 compared to $43,527) and a higher dentist rate (85 per 100,000 compared to 52 per 100,000). On all levels, Hawaii has better numbers relating to the variables in our model; it has higher percentages relating to negative coefficients and lower percentages relating to positive coefficients.

Overall, we find that SDOH can explain a substantial amount of the variation in state levels of edentulism, particularly relating to education and smoking. Our findings on tobacco use and tooth loss are not surprising and the role of dentists in tobacco use has been a subject of recent research. Gajendra, McIntosh, and Ghosh (2023) [27] provided a detailed literature review that found that oral health providers, including dentists, should be a primary location for tobacco use prevention. However, their findings indicate that dentists should be better trained to have this discussion with patients, and dental professionals should be financially incentivized to address and manage the ill effects of tobacco on both oral health and overall health. More general state policies could include education outreach about the benefits of good oral care and improving health behaviors such as lowering smoking rates and the health behaviors that underlie diabetes.

States with large rural populations are more likely to have higher rates of edentulism, suggesting that rural health disparities remain an important policy priority in the US. Policymakers in these states should aim to improve access to oral health by focusing on the specific challenges we share in our findings. Our results show that states with higher dental visits also have better oral health, and we provide some evidence of the role of insurance in this regard, although dental insurance seems to be a bigger indicator than pct_medicareC.

Our finding that a higher dentist rate is related to higher rates of tooth loss in two models may have several explanations that warrant further investigation. First, it is unlikely that more dentists are causing more edentulism, and our study does not identify causal links. It may therefore be a case of reverse causation, in that dentists are choosing to practice in areas with higher edentulism rates. This could be the impact of public insurance funding changes (Medicare and Medicaid). Huh (2021) [28] and Huh and Lin (2025) [29] describe the ways that public health insurance expansions were designed to increase access to primary care services including dental care. These policy changes may have also resulted in providers, like dentists, choosing practice locations based on reimbursement and financial sustainability of their practices, as well as loan repayment programs such as the National Health Service Corp. Further investigation of how providers are reacting to these changes in public health policy in their practice location decisions would be a useful line of future research. Second, the dentist rate may not be a good indicator of access to dental care. Future research should endeavor to gain a better understanding of access to dental care beyond dentist rate and insurance coverage, such as location and time convenience.

There are important limitations to our findings that can be addressed in future research. Geospatial research must avoid the ecological fallacy or tendency to draw conclusions about individuals based on findings at the state or local level [8]. Our study makes no claims of individual risk factors, whether that is demographic or behavioral. Similarly, while we would endeavor to address the current and growing issue of racial and ethnic disparities in oral health outcomes [6,8], our research cannot provide insights on this challenging issue. We have highlighted some data limitations already, especially over dental insurance coverage (regional coverage, years), but there is also some temporal and age mismatch in some of our data. Our rate of edentulism is for state residents aged 65 or over, but some of our variables are for adults aged over 18, or different age groups. While dental health in older residents is the cumulative effects of events and behaviors over decades, it is not obvious that current dental visits by those aged 18 or over, for example, are a good indicator of available dental care when today’s older residents were younger. Moreover, our research design can only identify associations or correlations and makes no claims to causal effects. Future research could include identifying key states with differing oral health outcomes (for example, variances between Hawaii and West Virginia) and exploring county-level differences to develop local public policy initiatives based on social determinants of health, including racial and ethnic disparities.

## 5. Conclusions

The 2000 Surgeon General report stated that reasons for the variation in state edentulism were unknown [1]. We identify several state-level characteristics that are associated with this variation. Important indicators include behaviors such as visiting a dentist and smoking as well as a more educated citizenry. We also find some evidence that dental insurance availability is associated with better edentulism rates.

## Figures and Tables

**Table 1:** Data sources and characteristics.

Variable	Source	Target Population	Geography	Years Available
Edentulism rate	CDC	State population aged 65+	State	2012, 2014, 2016, 2018, 2020
pct_visited_dentist	CDC	State population aged 18+	State	2012, 2014, 2016, 2018, 2020
dentist_rate	CHR	State population	State	2012, 2014, 2016, 2018, 2020
pct_medicareC	CMS	Percent of state population that are Medicare Part C beneficiaries	State	2016, 2018, 2020
pct_dental_insurance	Blackwell [27] (CDC)	Adults aged 18–64	Census Regions	2014–2017
income (in $000’s)	CHR	Households	State	2012, 2014, 2016, 2018, 2020
pct_some_college	CHR	Adults 25–44	State	2012, 2014, 2016, 2018, 2020
pct_smoking	CHR	Adults 18+	State	2012, 2014, 2016, 2018, 2020
pct_diabetes	CHR	Adults 18+	State	2012, 2014, 2016, 2018, 2020
pct_white	CHR	Resident population	State	2012, 2014, 2016, 2018, 2020
pct_black	CHR	Resident population	State	2012, 2014, 2016, 2018, 2020
pct_hispanic	CHR	Resident population	State	2012, 2014, 2016, 2018, 2020
pct_female	CHR	Resident population	State	2012, 2014, 2016, 2018, 2020
pct_rural	CHR	State population	State	2012, 2014, 2016, 2018, 2020

**Table 2:** Descriptive statistics and correlations for all states for even years 2012 through 2020.

Variables	Mean	SD	(1)	(2)	(3)	(4)	(5)	(6)	(7)	(8)	(9)	(10)	(11)	(12)	(13)
(1) Edentulism rate	16.5	5.4													
(2) pct_visited_dentist	59.9	13.5	1												
(3) dentist_rate	66.2	14.7	0.63	1											
(4) pct_medicareC	5.6	2.5	0.04	0.14	1										
(5) pct_dental_insurance	50.9	4.4	0.63	0.38	−0.06	1									
(6) income (in $000’s)	58.7	11.2	0.65	0.77	0.04	0.41	1								
(7) pct_some_college	65.5	5.3	0.77	0.71	0.03	0.58	0.7	1							
(8) pct_smoking	17.5	3.5	−0.59	−0.55	0	−0.25	−0.75	−0.55	1						
(9) pct_diabetes	9.8	1.7	−0.67	−0.63	−0.04	−0.46	−0.61	−0.70	0.58	1					
(10) pct_white	68.7	16.1	0.05	−0.24	0	0.3	−0.30	0.09	0.41	−0.15	1				
(11) pct_black	11.1	10.6	−0.27	−0.12	−0.13	−0.18	−0.06	−0.12	0.14	0.51	−0.44	1			
(12) pct_hispanic	11.7	10.1	−0.03	0.15	−0.02	−0.17	0.16	−0.11	−0.53	−0.11	−0.64	−0.13	1		
(13) pct_female	50.6	0.8	−0.07	−0.11	−0.05	0.12	−0.08	−0.09	0.04	0.46	−0.24	0.72	−0.05	1	
(14) pct_rural	26.1	14.8	−0.37	−0.46	−0.09	−0.11	−0.54	−0.27	0.67	0.21	0.64	−0.14	−0.61	−0.17	1

**Table 3:** Regression results for all states for even years 2012 through 2020.

	(1)	(2)	(3)
pct_visited_dentist	−0.179	−0.022	−0.157
	(−0.295–−0.063)	(−0.033–−0.011)	(−0.271–−0.044)
dentist_rate	0.061	0.038	0.058
	(0.013–0.110)	(−0.012–0.087)	(0.010–0.105)
pct_medicareC	−0.064		−0.075
	(−0.197–0.069)		(−0.215–0.066)
dental_insurance		−0.178	−0.143
		(−0.301–−0.055)	(−0.239–−0.047)
income	−0.047	0.008	−0.023
	(−0.142–0.047)	(−0.088–0.104)	(−0.115–0.069)
pct_some_college	−0.267	−0.325	−0.233
	(−0.401–−0.133)	(−0.494–−0.156)	(−0.368–−0.099)
pct_smoking	0.263	0.193	0.311
	(0.058–0.469)	(0.022–0.363)	(0.118–0.505)
pct_diabetes	0.225	0.248	0.186
	(−0.107–0.557)	(−0.014–0.509)	(−0.140–0.511)
pct_white	0.079	0.115	0.092
	(0.039–0.120)	(0.070–0.160)	(0.052–0.133)
pct_black	0.060	0.091	0.045
	(−0.019–0.140)	(0.038–0.145)	(−0.034–0.124)
pct_hispanic	−0.000	0.024	0.008
	(−0.057–0.056)	(−0.034–0.081)	(−0.051–0.067)
pct_female	0.681	0.811	1.048
	(−0.110–1.472)	(0.188–1.433)	(0.297–1.798)
pct_rural	0.039	0.074	0.038
	(−0.001–0.079)	(0.038–0.109)	(−0.001–0.077)
year	−0.058	0.000	−0.101
	(−0.310–0.193)	(−0.179–0.179)	(−0.349–0.147)
Constant	112.289	−14.712	181.676
	(−378.529–603.108)	(−362.008–332.583)	(−307.243–670.595)
Overall R-squared	0.86	0.82	0.86
Observations	153	255	153
Number of states	51	51	51

Note: Robust 95% confidence intervals clustered at the state level in parentheses.

**Table 4:** Correlated random effects results.

	(FE)	(CRE) (xt_means)
pct_visited_dentist	−0.036	−0.213
	(−0.160–0.088)	(−0.457–0.031)
dentist_rate	0.155	−0.086
	(−0.177–0.487)	(−0.434–0.263)
pct_medicareC	−0.128	0.061
	(−0.680–0.424)	(−0.564–0.686)
dental_insurance	−0.089	Omitted
	(−0.206−0.028)	
income	0.031	−0.038
	(−0.160–0.221)	(−0.261–0.185)
pct_some_college	0.362	−0.549
	(−0.114–0.839)	(−1.036–−0.061)
pct_smoking	0.019	0.296
	(−0.321–0.360)	(0−0.192–0.785)
pct_diabetes	0.014	0.488
	(−0.326–0.354)	(−0.188–1.163)
pct_white	−0.327	0.422
	(−1.552–0.898)	(−0.802–1.646
pct_black	0.151	−0.128
	(−1.259–1.560)	(−1.525–1.269)
pct_hispanic	−0.459	0.467
	(−2.185–1.267)	(−1.256–2.190)
pct_female	1.367	−0.455
	(−5.323–8.056)	(−7.322–6.431)
pct_rural	−0.147	0.179
	(−0.549–0.256)	(−0.226–0.583)
year	−0.694	Omitted
	(−1.540–0.152)	
Constant	1379.647	
	(−339.100–3098.394)	
Overall R-squared	0.88	0.82
Observations	153	153
Number of states	51	51

Note: Robust 95% confidence intervals clustered at the state level in parentheses. Mundalk test (tx_means = 0): chi2(12) = 23.5; *p* = 0.0236.

## Data Availability

Data supporting reported results can be found in the following publicly archived data sets. https://www.cdc.gov/oral-health-data-systems/oral_health_data/oral-health-dashboard.html accessed 20 September 2023; https://www.countyhealthrankings.org/ accessed 6 September 2023; https://data.cms.gov/summary-statistics-on-use-and-payments/medicare-geographic-comparisons/medicare-advantage-geographic-variation-national-state/data accessed 30 May 2025.
